# Midostaurin potentiates rituximab antitumor activity in Burkitt’s lymphoma by inducing apoptosis

**DOI:** 10.1038/s41419-018-1259-5

**Published:** 2018-12-18

**Authors:** Xiaowen Ge, Jianfeng Chen, Ling Li, Peipei Ding, Qi Wang, Wei Zhang, Luying Li, Xinyue Lv, Danlei Zhou, Zhengzeng Jiang, Haiying Zeng, Yifan Xu, Yingyong Hou, Weiguo Hu

**Affiliations:** 1 Department of Pathology, Zhongshan Hospital, Fudan University, Shanghai, China; 2 Fudan University Shanghai Cancer Center and Institutes of Biomedical Sciences; Shanghai Medical College, Fudan University, Shanghai, 200032 China

## Abstract

An intensive short-term chemotherapy regimen has substantially prolonged the overall survival of Burkitt’s lymphoma (BL) patients, which has been further improved by addition of rituximab. However, the inevitable development of resistance to rituximab and the toxicity of chemotherapy remain obstacles. We first prepared two BL cell lines resistant to rituximab-mediated CDC. Using a phosphorylation antibody microarray, we revealed that PI3K/AKT pathway contained the most phosphorylated proteins/hits, while apoptosis pathway that may be regulated by PKC displayed the greatest fold enrichment in the resistant cells. The PI3K/AKT inhibitor IPI-145 failed to reverse the resistance. In contrast, the pan-PKC inhibitor midostaurin exhibited potent antitumor activity in both original and resistant cells, alone or in combination with rituximab. Notably, midostaurin promoted apoptosis by reducing the phosphorylation of PKC and consequently of downstream Bad, Bcl-2 and NF-κB. Therefore, midostaurin improved rituximab activity by supplementing pro-apoptotic effects. In vivo, midostaurin alone powerfully prolonged the survival of mice bearing the resistant BL cells compared to rituximab alone treatments. Addition of midostaurin to rituximab led to dramatically improved survival compared to rituximab but not midostaurin monotherapy. Our findings call for further evaluation of midostaurin alone or in combination with rituximab in treating resistant BL in particular.

## Introduction

Burkitt’s lymphoma (BL), a highly aggressive non-Hodgkin’s B-cell lymphoma, accounts for 3–5% of lymphoma cases in all age groups and 40–50% of all childhood lymphomas^1^. Adult BL patients have shown a poor response to a CHOP (cyclophosphamide, doxorubicin, vincristine and prednisolone)-based regimen, with 2-year and 5-year overall survival (OS) rates of approximately 50–65%, decreasing to less than 30% with bone marrow or central nervous system involvement^2,3^. In contrast, an intensive short-term chemotherapy regimen has substantially improved the survival rates to greater than 90% in childhood BL patients^4,5^. Similar regimens in adult BL patients have achieved advances in outcomes, with OS rates exceeding 70%^6–9^. Despite the success of these regimens, further progress is required to achieve a therapeutic strategy that can reduce toxicity and overcome drug resistance in currently incurable patients.

The combination of rituximab with CHOP chemotherapy (R-CHOP) has improved overall survival by at least 20% in cases of diffuse large B-cell lymphoma (DLBCL)^10^. Similarly, many single-arm clinical trials have confirmed the effect of adding rituximab to the intensive short-term chemotherapy regimens for BL^11–15^. A recent phase III clinical trial has shown that addition of rituximab to chemotherapy achieved better 3-year event-free survival (75% vs 62%, *P* = 0.024) and 3-year OS (83% vs 70%, *P* = 0.011) rates than chemotherapy alone^16^. Therefore, addition of rituximab is highly anticipated in the future design of anti-BL regimens, and the subsequent development of resistance to rituximab may be predictable as it has occurred in DLBCL^17^.

By binding to the membrane protein CD20, rituximab destroys lymphocytes mainly via antibody-dependent cellular cytotoxicity (ADCC) and complement-dependent cytotoxicity (CDC)^17–19^. The resistance to ADCC probably results from the intrinsic polymorphism of the IgG Fc receptor *FcgammaRIIIa* gene^20^, whereas the resistance to CDC can most likely be attributed to the down-regulation of CD20 expression and the elevated expression of membrane complement regulatory proteins (mCRPs), especially CD59 expression^17,21,22^. However, many studies have revealed that rituximab fails to induce apoptosis to any detectable extent in B-cell lymphoma, including in BL cells^23–30^. Therefore, the development of a pro-apoptotic agent to combine with rituximab is a rational approach to achieving either high anti-cancer efficacy with rituximab or overcoming the resistance to rituximab. To identify such an alternative therapeutic approach, we prepared two BL cell lines resistant to rituximab-mediated CDC, interrogated the signaling pathways related to the development of resistance, and evaluated the effect of pathway inhibitors on antitumor activity and overcoming resistance.

## Materials and methods

### Cell culture and reagents

Two BL cell lines, Raji and Ramos, were purchased from American Type Culture Collection (Manassas, VA) and were maintained in RPMI-1640 medium supplemented with 10% fetal bovine serum (GIBCO BRL, Grand Island, NY) and 1% penicillin/streptomycin (Ambion, Austin, TX).

As a complement resource, normal human serum (NHS) was pooled from 10 healthy persons, aliquoted and stored at −80 °C until use. The phosphoinositide 3-kinase (PI3K) inhibitor IPI-145^31^ and the protein kinase C (PKC) inhibitor midostaurin were purchased from Selleck Chemicals (Houston, TX), and dissolved in dimethyl sulfoxide (DMSO) in use. Consequently, same volume DMSO was used as control.

### Generation of rituximab-resistant BL cells

We developed Raji and Ramos cells that were resistant to rituximab-mediated CDC as previously described^32^. Briefly, the original Raji or Ramos cells were treated with escalating rituximab (Roche, Basel, Switzerland) concentrations from 4 or 40 μg/mL to 32 or 640 μg/mL, respectively, in the presence of 20% NHS. The resulting resistant cells were termed Raji32 and Ramos640, respectively. These cells were treated with 32 μg/mL or 640 μg/mL rituximab, respectively, and 20% NHS every 21 days to maintain resistance. The CDC effect was assessed by fluorescence-activated cell sorting (FACS) analysis to detect propidium iodide-positive cells.

### Immunoblotting assay

We performed immunoblotting assays according to the standard protocol using the antibodies shown in Table S1.

### FACS analysis

After washing with phosphate-buffered saline (PBS), cells were incubated with fluorescein-conjugated antibodies for 30 min and then rinsed and resuspended in PBS. Flow cytometric analysis was performed on a Cytomics FC500 MPL machine (Beckman Coulter, Brea, CA) and analyzed with the FlowJo software (Ashland, OR). We performed cell sorting with a MoFlo XDP instrument (Beckman Coulter, Brea, CA) based on fluorescence.

Apoptosis analysis was performed using the PE Annexin V Apoptosis Detection Kit (BD Pharmingen, San Diego, CA) according to the manufacturer’s instructions.

### CytoTox-Glo™ cytotoxicity assay

We used the CytoTox-Glo™ cytotoxicity assay kit (Promega, Madison, WI) to detect cytotoxicity. The cells were pretreated with rituximab and 20% NHS in the presence or absence of midostaurin for 48 h before assays. Cytotoxicity was calculated according to the formula: cytotoxicity (%) = dead cell luminescence/total luminescence × 100%.

### Antibody microarray for profiling phosphorylation

Protein phosphorylation was profiled using a phosphorylation-profiling antibody microarray (Full Moon Microsystems, Catalog No. CSP100, Sunnyvale, CA) containing 269 antibodies against 131 protein phosphorylation sites; the procedure was performed by Wayen Biotechnology (Shanghai, China) according to the established protocol. Differentially phosphorylated proteins with a fold change >1.5 were screened by DAVID (Database for Annotation, Visualization and Integrated Discovery) to performed functional annotation^33^. The interaction network among PKC subunits and the differentially phosphorylated proteins was analyzed using Ingenuity Pathway Analysis (IPA, QIAGEN, Dusseldorf, Germany).

### RNA sequencing

RNA sequencing (RNA-seq) was performed as previously described^34^. Briefly, total RNA was extracted and pooled separately from three different passages of Ramos and Ramos640 cells. The RNA quality was determined with a Bioanalyzer 2200 (Agilent Technologies, Santa, Clara, CA). RNA with an RNA integrity number (RIN) >8.0 was considered acceptable for complementary DNA (cDNA) library construction. The RNA-seq was conducted by Shanghai Novelbio, Ltd. cDNA libraries were constructed using the Ion Total RNA-seq Kit v2.0 (Life Technologies, Gaithersburg, MD) and were then processed for RNA sequencing. Before the read mapping, clean reads were obtained from the raw reads by removing adaptor sequences, and these reads were then aligned to the human genome (version: GRCh37 NCBI) using the MapSplice program (v2.1.6). After the significance and false discovery rate (FDR) analyses were performed under the following criteria: (1) fold change >1.5 or <0.667 and (2) FDR <0.05, we applied the DEseq algorithm to filter the differentially expressed genes.

### Gene set enrichment analysis

Using gene set enrichment analysis (GSEA) software (the Broad Institute at MIT), we performed a GSEA to identify the functions of the differentially expressed genes obtained from the RNA-seq^35,36^. The pre-ranked version of the software was used to identify significantly enriched pathways, and enriched pathways with an FDR <0.25 were considered significant. The anti-apoptotic TNFs/NF-κB/Bcl-2 pathway gene set used in this study consisted of 42 genes from the ‘apoptosis and survival anti-apoptotic TNFs/NF-κB/Bcl-2 pathway SuperPath’ in the PathCards pathway unification database (Version 4.6.0.37, Weizmann Institute of Science). The p53 pathway gene set used in this study consisted of 132 genes from the ‘p53 pathway (RnD) SuperPath’ in the PathCards pathway unification database.

### Plasmid construction and lentiviral transduction

The Coding Sequence (CDS) of the *firefly luciferase* gene was obtained by PCR amplification from the pGL3-Basic plasmid and inserted into the pCDH cDNA cloning and expression lentivector. Primers for the *firefly luciferase* CDS amplification were as follows: forward primer 5′-ATGGAAGACGCCAAAAACATAAAG-3′, reverse primer 5′-TTACACGGCGATCTTTCCGCCCTT-3′. The pCDH plasmid was co-transfected with the pMD.2G and psPAX2 plasmids into 293FT cells to generate a *firefly luciferase* overexpression lentivirus. The lentivirus was subsequently added to the culture medium of Raji32 cells for 48 h of incubation. All the cells transfected with the lentivirus in this study were sorted based on green fluorescent protein using a MoFlo XDP instrument (Beckman Coulter, Brea, CA) and were termed Raji32-Luc cells.

### Xenograft model

The 8-week-old female SCID (severe combined immunodeficiency) mice were purchased from SLAC Laboratory Animal Co. (Shanghai, China). Raji32-Luc cells were resuspended in PBS and then injected intraperitoneally at 1.5 × 10^7^ cells per mouse. The mice were divided into 4 groups (7 mice per group) based on the administered drugs, i.e., saline, rituximab, midostaurin and rituximab plus midostaurin. Rituximab was intraperitoneally injected at 118.4 mg/kg on days 8, 12 and 16, and midostaurin was administered by gavage at 20 mg/kg on days 8, 9, 10, 11, 12, 13, 14, 15, 16, 17, 18, 19, 20 and 21 after implantation. Saline was administered in the same way as rituximab with the same injection volume. Tumor growth was monitored by bioluminescence at 50, 70 and 90 days after implantation. For the in vivo luminescence imaging, D-luciferin (Promega, Madison, WI) was intraperitoneally injected (150 mg/kg). After 10 min, the mice were anesthetized by intraperitoneal injection with pentobarbital (50 mg/kg), and bioluminescence was then visualized using an In-Vivo MS FX PRO system (Bruker, Billerica, MA). The survival time of each mouse was recorded until 120 days. All the animal experiments were conducted in strict accordance with experimental protocols approved by the Animal Ethics Committee at Shanghai Medical School, Fudan University.

### Statistics

The data are presented as the mean ± SD unless otherwise specified. The significance of differences between two groups was determined using the two-tailed Student’s *t*-test for unpaired data. For the total photon flux in the animal models, the significance of differences was determined using the one-tailed Mann–Whitney test. We applied the Mantel–Cox test to compare the survival rates of two groups of xenograft mouse models. In all analyses, *P* < 0.05 was considered statistically significant.

## Results

### Reduced CD20 and elevated CD59 expression levels led to the resistance of BL cells to rituximab-mediated CDC

**Fig. 1:**
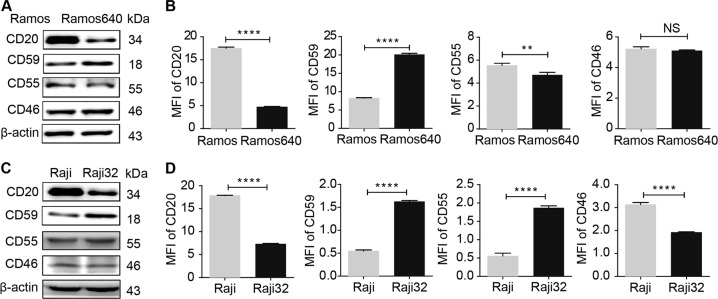
The expression levels of CD20 and mCRPs in resistant BL cells. **a**–**d** Compared to the original cells, CD20 expression was reduced, while CD59 expression was elevated in the resistant Ramos640 (**a**, **b**) and Raji32 (**c**, **d**) cells as determined by western blot analysis (**a**, **c**) and FACS (**b**, **d**). However, CD55 expression was slightly reduced in the resistant Ramos640 cells (**a**, **b**), but significantly increased in the resistant Raji32 cells (**c**, **d**). In addition, CD46 expression was only decreased in the resistant Raji32 cells (**c**, **d**) but not in the Ramos640 cells (**a**, **b**). The data are presented as the means ± SD, *n* = 3, NS  no significance, **P < 0.01, and ****P < 0.0001

The intrinsic features of immune cells precipitate the resistance to rituximab-mediated ADCC^20^, while apoptosis plays a negligible role in rituximab antitumor activity. Therefore, we generated two BL cell lines, Ramos640 and Raji32, that were resistant to rituximab-mediated CDC at rituximab concentrations of 640 and 32 μg/mL, respectively. Using immunoblotting (Fig. 1a, c) and FACS (Fig. 1b, d) assays, we found that CD20 expression decreased while CD59 expression increased in both resistant cell lines compared to the expression levels in the corresponding original cells (Fig. 1). However, the expression levels of two other mCRPs were not consistent between the two resistant cell lines. CD55 expression was reduced in Ramos640 cells but was increased in Raji32 cells, and CD46 expression was unchanged in Ramos640 cells but was reduced in Raji32 cells (Fig. 1). These results are consistent with previous reports showing that reduced CD20 and elevated CD59 levels contribute to the development of resistance to rituximab-mediated CDC^17,21,22^. Considerable efforts have been made to improve rituximab therapeutic efficacy by increasing CD20 expression, for example, by administering the histone deacetylase inhibitor trichostatin A^37^ or synthetic CpG oligodeoxynucleotides^38^ or by inhibiting CD59 function with a modified monoclonal antibody^39,40^ or the bacterial toxin-derived ILYd4^32^. However, among these approaches, only CpG oligodeoxynucleotides were further evaluated in a clinical trial (phase I) to determine their safety in B-cell non-Hodgkin lymphoma (NHL) patients^41^, and no further clinical trials have been reported. Therefore, alternative therapeutic strategies are still required.

### PI3K/Akt pathway was strongly enriched in the resistant cells, but its inhibition failed to reverse the resistance

**Fig. 2:**
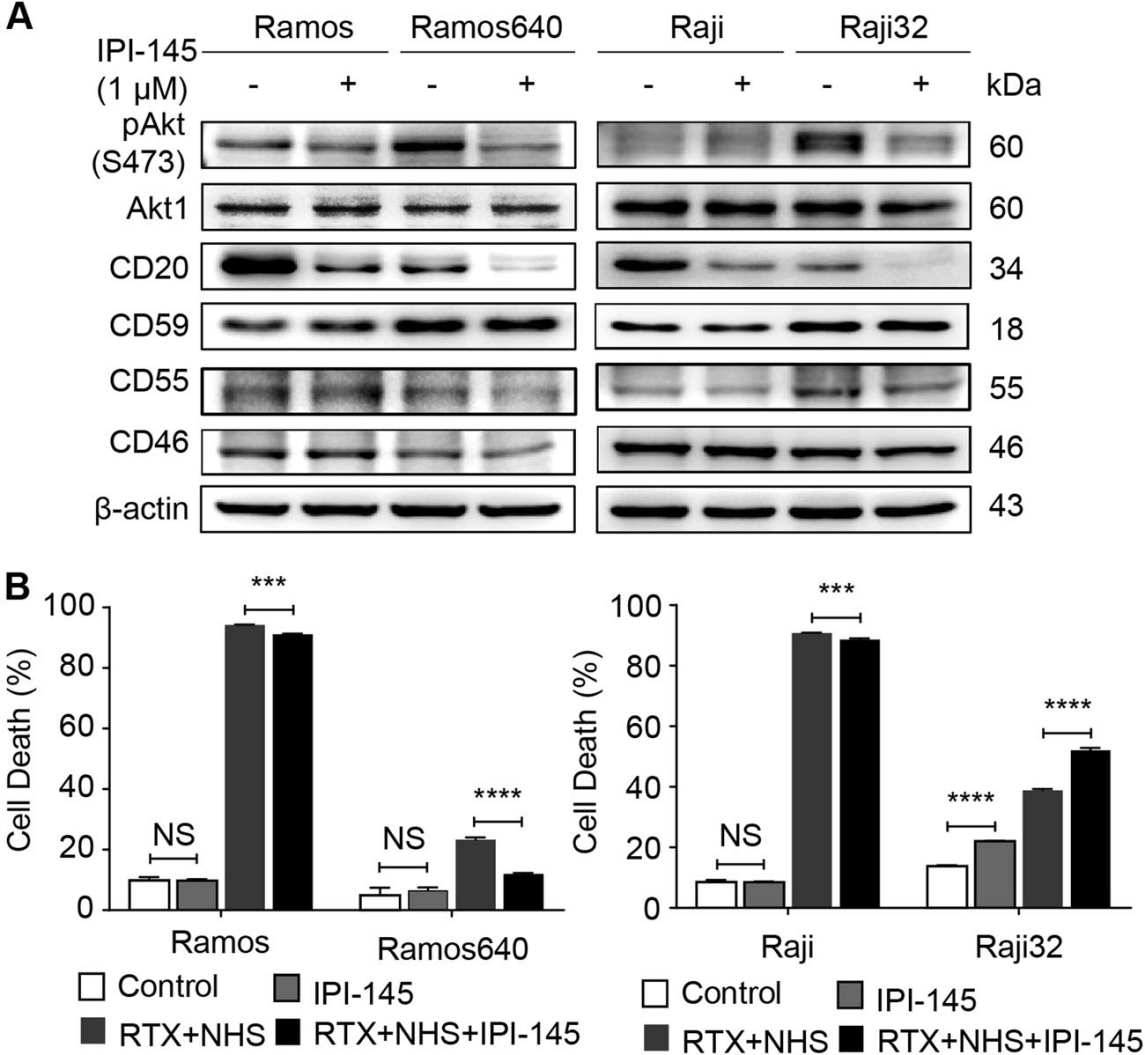
PI3K/Akt inhibition failed to enhance the susceptibility of BL cells to rituximab-mediated CDC. **a** The phosphorylation levels of Akt (S473) increased in the resistant Ramos640 and Raji cells compared to the corresponding original cells, which could be suppressed by treatment with the PI3K inhibitor IPI-145, leading to reduced expression of CD20 and CD55. **b** The effect of combining IPI-145 with rituximab on rituximab-mediated CDC. IPI-145 alone failed to increase cell death in all the original and resistant cells except for the Raji32 cells. The addition of IPI-145 to rituximab also failed to increase rituximab-mediated CDC in the Ramos and Raji cells, but not in the Raji32 cells. In contrast, this combination treatment significantly increased rituximab-mediated CDC in the Raji32 cells. The data are represented as the means ± SD, *n* = 3, NS no significance, ***P < 0.001, ****P < 0.0001. RTX rituximab, NHS normal human serum

Considering the negligible effect of rituximab on inducing apoptosis in B-NHL, addition of a pro-apoptotic agent to rituximab therapy would be an ideal strategy. Therefore, an antibody microarray for phosphorylation profiling was used to identify the functional proteins and signaling pathways activated in Ramos640 cells. Fluorescence analysis revealed that the phosphorylation levels of 35 proteins were up- (31/35) or down-regulated (4/35) by greater than 1.4-fold in Ramos640 cells compared to the levels in Ramos cells (Supplementary Data 1). KEGG (Kyoto Encyclopedia of Genes and Genomes) signaling pathway analysis of these proteins demonstrated that PI3K/AKT signaling pathway contained the most phosphorylated proteins/hits, with 13, while apoptosis pathway displayed the greatest fold enrichment, at 47.76 (Table 1). Next, the PI3K inhibitor IPI-145 was used to determine whether PI3K inhibition could increase the susceptibility of resistant BL cells to rituximab treatment. We first confirmed that PI3K/Akt was activated in Ramos640 and Raji32 cells compared to the corresponding original cells and that IPI-145 effectively impaired the phosphorylation of Akt (Fig. 2a). However, CD20 expression was strongly suppressed by IPI-145 treatment in both original and resistant cells, whereas the expression levels of three mCRPs CD59, CD55 and CD46 were nearly unchanged (Fig. 2a). These outcomes subsequently resulted in no effect or even a slightly negative effect of IPI-145 on the enhancement of rituximab-mediated CDC in the original and resistant cells, with the exception of Raji32 cells (Fig. 2b). In the Raji32 cells, IPI-145 alone or in combination with rituximab slightly enhanced cell death compared to that observed in the control or rituximab alone treatments, respectively (Fig. 2b, right panel). These results suggest that PI3K/Akt might not be a valuable drug target for facilitating rituximab treatment.

**Table 1:** Enriched pathways in Ramos640 vs Ramos cells based on an antibody microarray for profiling phosphorylation

KEGG pathway	*P* value	Fold enrichment	Count	Genes
hsa04151: PI3K-Akt signaling pathway	2.47E−15	18.60	13	*CDKN1A, PTK2, CDKN1B, BCL2, CREB1, TP53, BAD, BCL2L1, ITGB3, PTEN, MYC, CHUK, AKT2*
hsa05200: Pathways in cancer	1.24E−12	15.07	12	*CDKN1A, PTK2, CDKN1B, JUN, BCL2, TP53, BAD, BCL2L1, PTEN, MYC, CHUK, AKT2*
hsa04012: ErbB signaling pathway	5.35E−09	39.71	7	*CDKN1A, PTK2, CDKN1B, JUN, BAD, MYC, AKT2*
hsa05169: Epstein-Barr virus infection	1.59E−08	20.78	8	*CDKN1A, CDKN1B, JUN, BCL2, TP53, MYC, CHUK, AKT2*
hsa04210: Apoptosis	6.01E−08	47.76	6	*BCL2, TP53, BAD, BCL2L1, CHUK, AKT2*
hsa04510: Focal adhesion	9.41E−07	16.77	7	*PTK2, JUN, BCL2, BAD, ITGB3, PTEN, AKT2*
hsa05206: microRNAs in cancer	6.28E−06	12.12	7	*CDKN1A, CDKN1B, BCL2, TP53, ITGB3, PTEN, MYC*
hsa05202: Transcriptional misregulation in cancer	8.80E−06	17.63	6	*CDKN1A, PTK2, CDKN1B, TP53, BCL2L1, MYC*
hsa05205: Proteoglycans in cancer	2.06E−05	14.81	6	*CDKN1A, PTK2, TP53, ITGB3, MYC, AKT2*
hsa05203: Viral carcinogenesis	2.32E−05	14.45	6	*CDKN1A, CDKN1B, JUN, CREB1, TP53, BAD*

### The PKC-mediated apoptosis pathway was highly activated in resistant cells

**Fig. 3:**
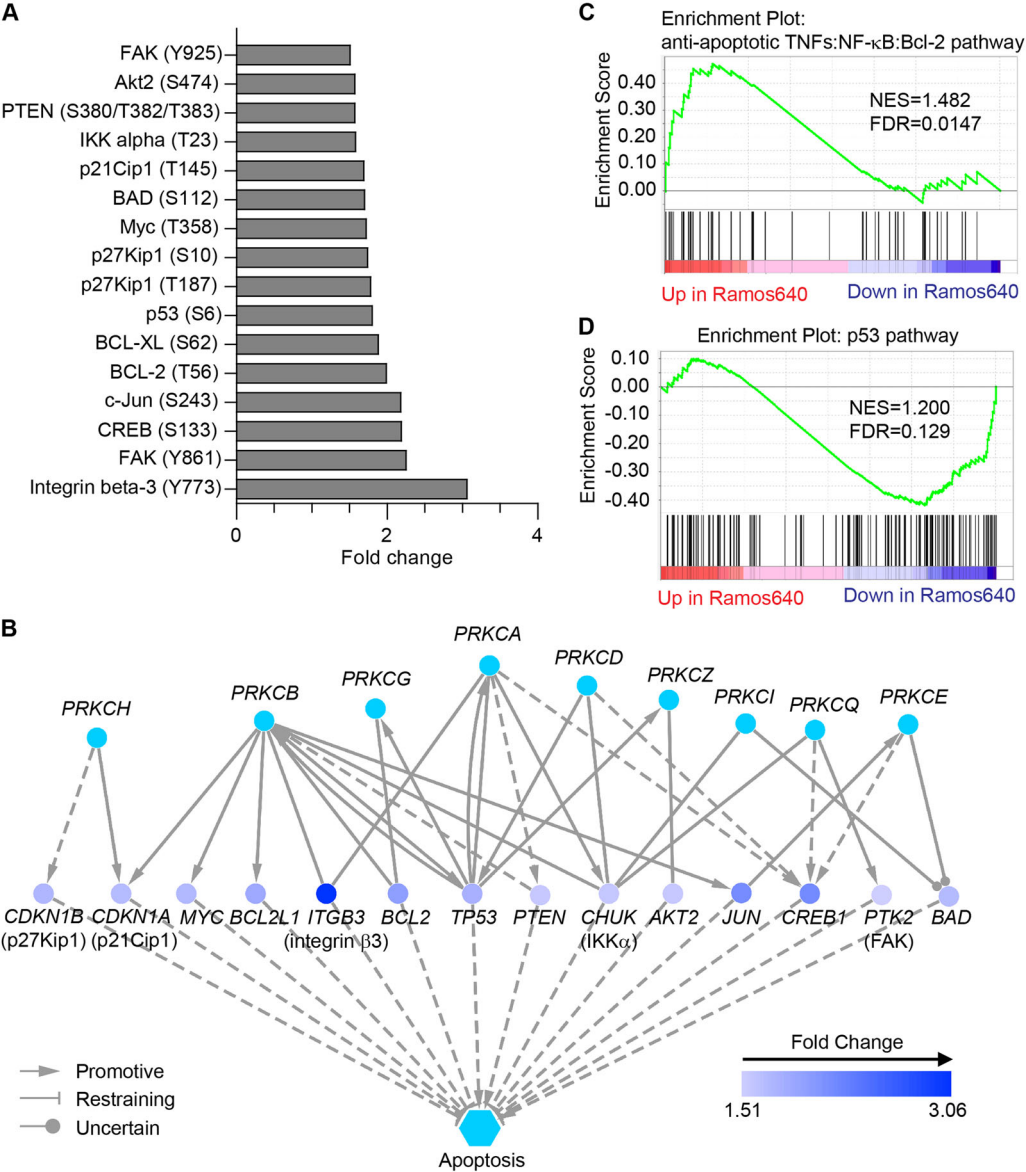
Bioinformatics analysis revealed the anti-apoptotic effect regulated by PKC signaling in Ramos640 cells. **a** Phosphorylated proteins/hits up-regulated by greater than 1.5-fold in an antibody microarray for profiling phosphorylation. **b** IPA: the distinct PKC isoforms regulate apoptosis via the up-regulated proteins/hits in (**a**). **c**, **d** GSEA for TNFs/NF-κB/Bcl-2 (**c**) or p53 (**d**) signatures in comparing the RNA-Seq data of the Ramos and Ramos640 cells. Results with an FDR < 0.25 were considered to be significant. Solid lines represent direct regulation, and dotted lines represent indirect regulation

Although apoptosis pathway was identified as the most enriched in the antibody microarray, only six proteins were included in this pathway (Table 1), which hindered the isolation of the pathway modulators. Therefore, we narrowed the scope of the investigation by elevating the fold change of up- and down-regulated phosphorylated proteins/sites to 1.5 in the phosphorylation-profiling antibody microarray. The results revealed that a total of 16 proteins/sites were up-regulated by greater than 1.5-fold (Fig. 3a, and Supplementary Data 1). Notably, all of these up-regulated proteins/sites were determined to be involved in apoptosis according to the IPA (Fig. 3b). More importantly, the IPA also revealed that PKC signaling regulates all of these up-regulated proteins (Fig. 3b), although the detection of PKC phosphorylation was not included in the antibody microarray set used to profile phosphorylation. Using RNA-seq assay, we further found that the expression of genes in TNFs/NF-κB/Bcl-2 pathway was strongly elevated, while the expression of genes in p53 pathway was significantly reduced in Ramos640 cells compared to the levels in Ramos cells based on the GSEA (Fig. 3c, d, and Supplementary Data 2). This analysis also indicated that the anti-apoptotic genes were up-regulated, while the pro-apoptotic genes were down-regulated. Therefore, these data suggested that PKC phosphorylated multiple downstream proteins, leading to the anti-apoptotic effect in Ramos640 cells.

### The pan-PKC inhibitor midostaurin strongly promoted apoptosis

**Fig. 4:**
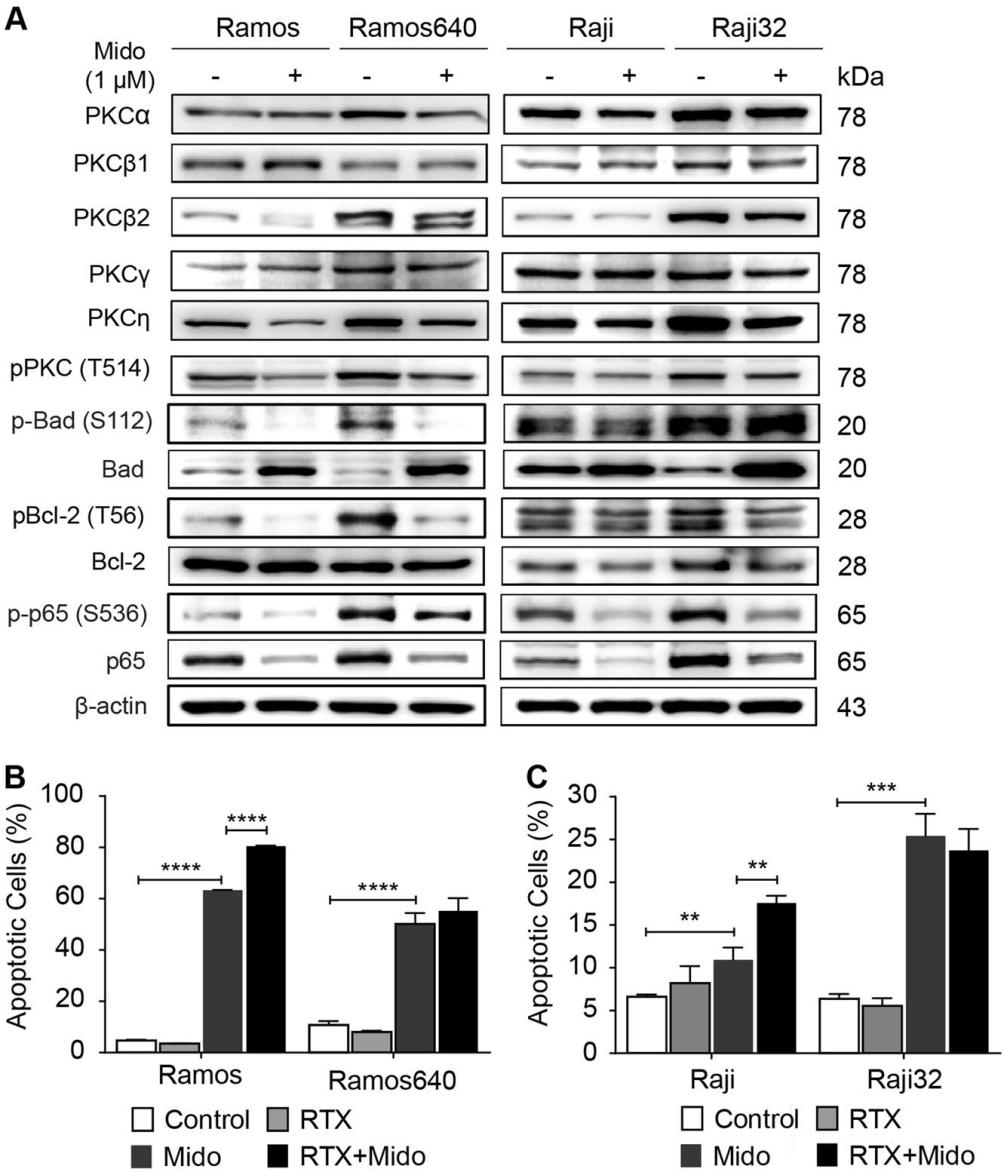
PKC inhibition strongly induced apoptosis in both the original and resistant BL cells. **a** The indicated PKC isoforms were up-regulated in the resistant BL cells, leading to elevated levels of PKC phosphorylation and the activation of downstream anti-apoptotic proteins. **b**, **c** The pan-PKC inhibitor midostaurin potently induced apoptosis in the original and resistant Ramos (**b**) and Raji (**c**) cells, and the addition of rituximab to midostaurin significantly enhanced the pro-apoptotic effect in the Ramos640 cells. The data are presented as the means ± SD, *n* = 3, **P < 0.01, ***P < 0.001, ****P < 0.0001. RTX rituximab (640 or 32 μg/mL for Ramos or Raji, respectively), Mido midostaurin (1 μM)

We compared the expression levels of five PKC isoforms in the original and resistant BL cells, and found that PKCα/β2/γ/η in Ramos640 and PKCα/β1/β2/η in Raji32 cells were overexpressed compared to the levels in the corresponding original cells. More importantly, the phosphorylation levels of PKC, which were detected by the sole commercially available antibody recognizing all PKC isoforms, were increased in both resistant cell lines (Fig. 4a). Further, we detected the efficacy of midostaurin in regulating PKC phosphorylation. Midostaurin is a multi-kinase inhibitor that was initially targeted toward PKC and is currently approved for acute myelogenous leukemia (AML) with an Fms-like tyrosine kinase 3 (FLT3) mutation and advanced systemic mastocytosis (SM)^42,43^. We observed that midostaurin suppressed the phosphorylation levels of PKC in both resistant BL cell lines and original Ramos cells but not original Raji cells and that this kinase inhibitor reduced the expression levels of PKCβ2/η in Ramos cells, of PKCα/η in Ramos640 cells and of PKCβ1/η in Raji32 cells (Fig. 4a).

Next, we verified the phosphorylation levels of several PKC downstream signaling molecules, including Bad, Bcl-2 and NF-κB subunit p65. We first found that the phosphorylated levels of these molecules were significantly elevated (Fig. 4a). Furthermore, the expression levels of Bad were decreased, while those of p65 were increased in both resistant cell lines (Fig. 4a). All of the above changes may contribute to anti-apoptosis to a considerable degree, thus leading to the development of rituximab resistance in Ramos640 and Raji32 cells. In addition, midostaurin suppressed the phosphorylation levels of Bad, Bcl-2 and p65 in all the original and resistant cells (Fig. 4a). Moreover, midostaurin increased or reduced the expression of Bad or p65, respectively, in all four original and resistant cell lines and reduced the expression of Bcl-2 only in the original and resistant Raji cells (Fig. 4a). Therefore, midostaurin inhibited PKC and its subsequent downstream signaling molecules, which may lead to the pro-apoptotic effect in both Ramos and Raji cells to varying degrees. Among the cell lines, Raji cells appeared to be more resistant to midostaurin-mediated PKC inhibition than Ramos cells because midostaurin failed to suppress PKC phosphorylation.

Furthermore, we assessed the pro-apoptotic effects of midostaurin alone or in combination with rituximab on the original and resistant BL cells. As we expected, treatment with rituximab alone failed to induce apoptosis in all four original or resistant BL cell lines (Fig. 4b, c). However, compared to the culture medium control, midostaurin alone dramatically induced cell apoptosis in Ramos (from 4.7 to 63.3%), Ramos640 (from 10.8 to 50.2%) and Raji32 (from 6.4 to 25.4%) cells and slightly increased apoptosis (from 6.6% to 10.9%) in Raji cells, although the *P* value did reach statistical significance (*P* = 0.0038) (Fig. 4b, c). These results also functionally indicated that, in general, Raji cells were more resistant to midostaurin-induced apoptosis than Ramos cells. In addition, the combination of midostaurin with rituximab resulted in slight pro-apoptotic effects only in the original but not in the resistant Ramos and Raji cell lines (Fig. 4b,c), further supporting the conclusion that rituximab induces a negligible pro-apoptotic effect, if any.

### Midostaurin additively enhanced the susceptibility of resistant BL cells to rituximab-mediated CDC

**Fig. 5:**
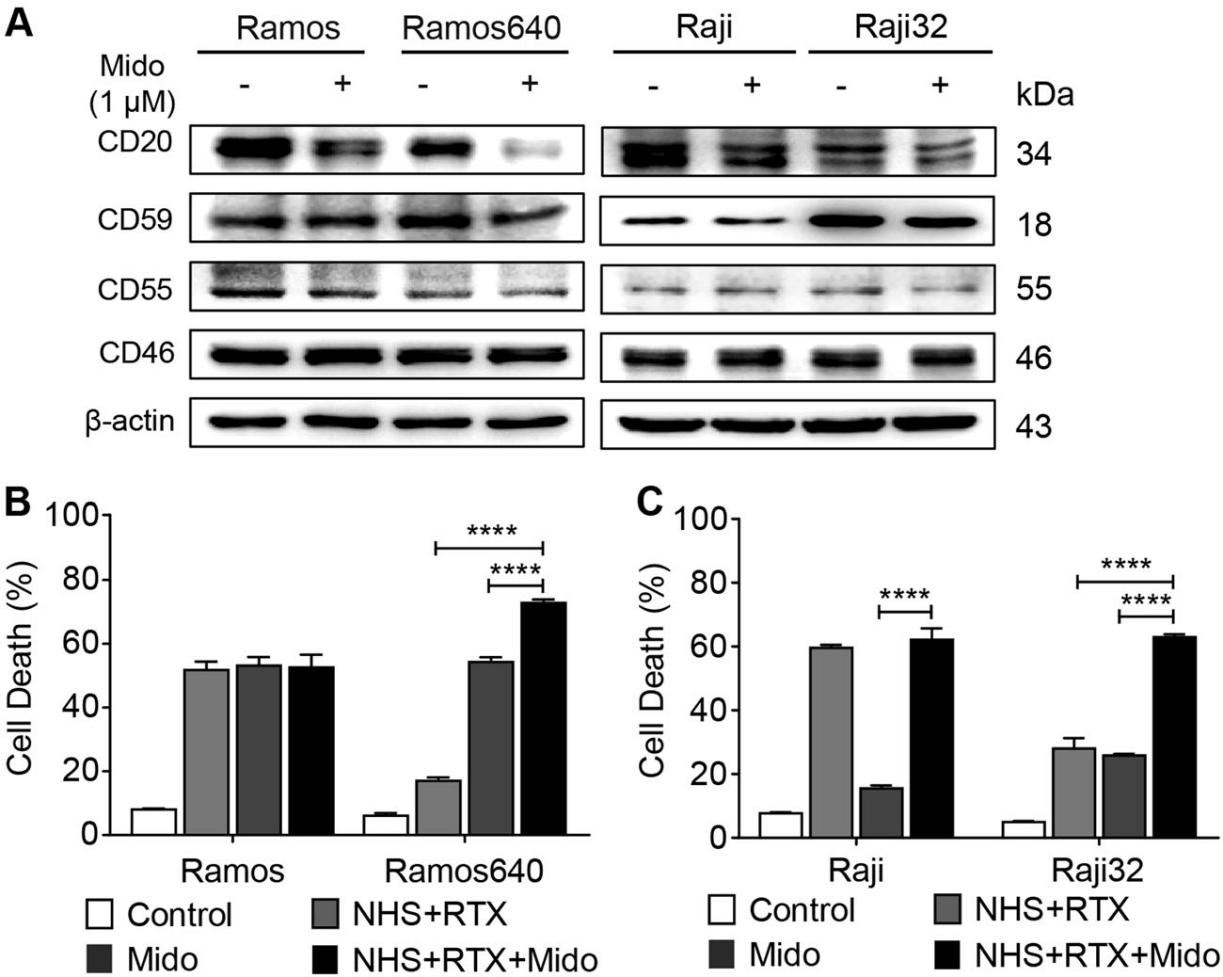
Midostaurin additively enhanced rituximab-mediated CDC in resistant BL cells. **a** The PKC inhibitor significantly reduced CD20 expression in both the original and resistant cells and CD59 expression mainly in the resistant cells but did not alter the expression of CD55 or CD46 in the original or resistant cells. **b**, **c** The effect of the PKC inhibitor on rituximab-mediated CDC. The addition of the PKC inhibitor failed to enhance rituximab-mediated CDC in the original Ramos (**b**) and Raji (**c**) cells; however, the inhibitor additively enhanced CDC in the resistant Ramos640 (**b**) and Raji32 (**c**) cells. The data are presented as the means ± SD, *n* = 3, ****P < 0.0001. RTX rituximab (40 or 4 μg/mL for Ramos or Raji, respectively; or 640 or 32 μg/mL for Ramos640 or Raji32, respectively), NHS normal human serum (20%), Mido midostaurin (1 μM)

Rituximab-mediated CDC can be regulated by the expression of mCRPs, such as CD46, CD55 and especially CD59, in addition to CD20^17,22,44^. We observed that midostaurin reduced the expression levels of CD20 in all four BL cell lines and the levels of CD59 mainly in the resistant cells, while this inhibitor had no effect on the expression levels of CD55 or CD46 (Fig. 5a). These results suggested that midostaurin may hinder rituximab-mediated CDC due to the reduced expression of only CD20 in the original BL cells, while the effect of midostaurin on the resistant BL cells requires further examination due to the reduced expression of both CD20 and CD59. In addition, these results revealed a difference between the IPI-145 and midostaurin effects on regulating CD20 and CD59 expression, in which IPI-145 only reduced CD20 but not CD59 expression in the resistant BL cells (Fig. 2a).

While rituximab plus NHS potently induced CDC in the original Ramos and Raji cells, addition of midostaurin failed to enhance the susceptibility to rituximab-mediated CDC, although midostaurin promoted apoptosis (Fig. 5b, c). This result may be caused by the reduced CD20 expression and the already high cell death rate induced by treatment with rituximab alone. In contrast, addition of rituximab significantly enhanced the cytotoxic effect of midostaurin in both original BL cell lines (Fig. 5b, c), indicating that rituximab-mediated CDC dominated over midostaurin-induced apoptosis in the original BL cells. Notably, we found that the cytotoxic effect of the combined rituximab and midostaurin treatment appeared to be additive in the resistant Ramos640 and Raji32 cells. Rituximab and midostaurin induced 17.1 and 54.3% cell death, respectively, while the combination induced 72.8% cell death (Fig. 5b). Similarly, rituximab and midostaurin induced 28.0 and 25.8% cell death, respectively, while their combination induced 62.9% cell death (Fig. 5c). These results may be due to the distinct antitumor mechanisms of rituximab and midostaurin.

### Combination treatment of rituximab with midostaurin significantly suppressed tumor growth of the resistant cells

**Fig. 6:**
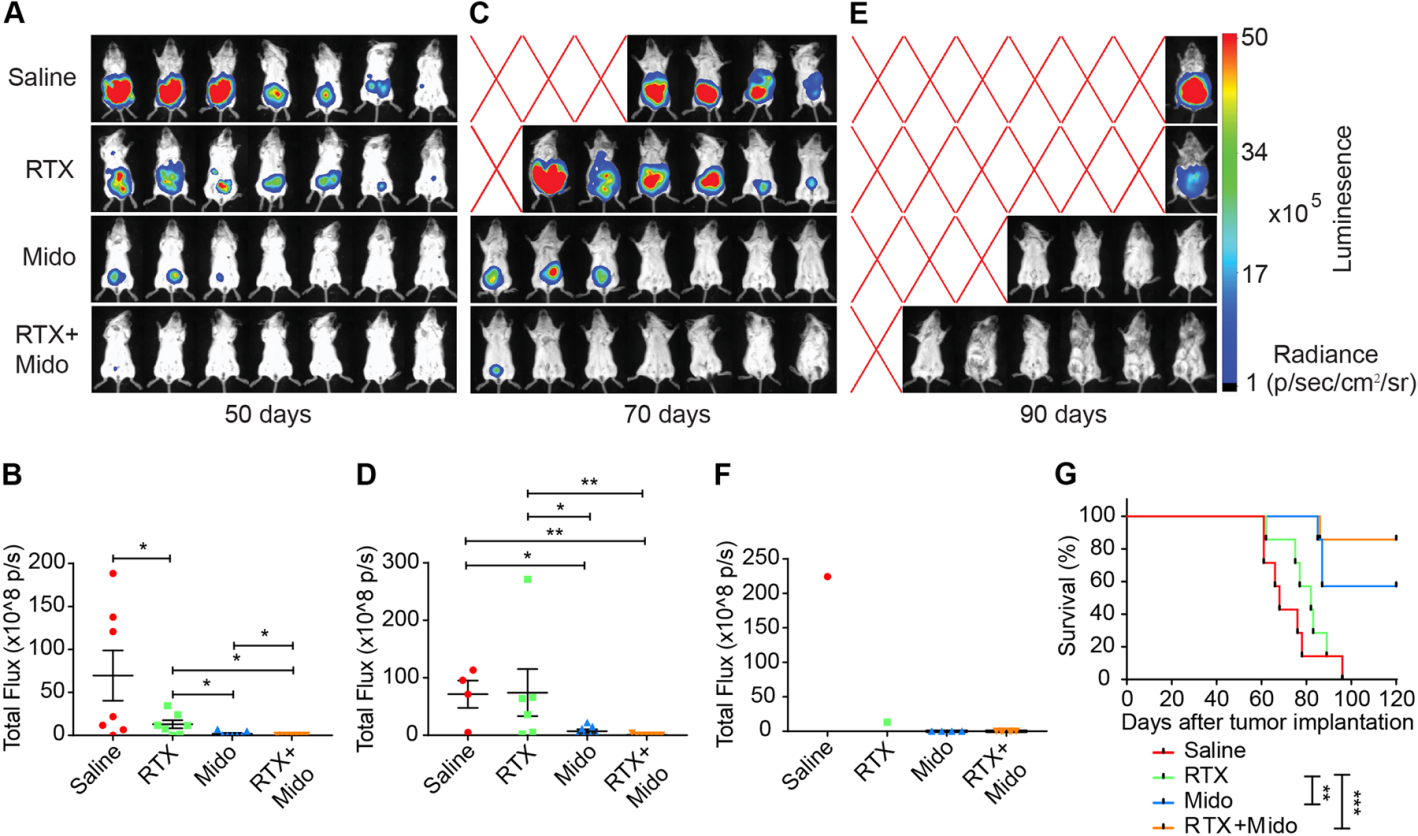
Combination treatment with midostaurin and rituximab significantly suppressed the tumor growth of the resistant Raji32 cells. Tumor masses are represented by the intensity of firefly luciferase activity (**a**, **c**, **e**), with the quantitative results of total photon flux shown in (**b**, **d**, **f**). **g** The survival curves of mice that were implanted with Raji32-Luc cells and treated with the indicated drugs. The data are presented as the means ± SEM (*n* = 7). *P < 0.05, **P < 0.01, ***P < 0.001. A red 'X' represents mouse death in (**c** and **e**). RTX rituximab (intraperitoneally injected at 118.4 mg/kg on days 8, 12 and 16), Mido midostaurin (administrated by gavage at 20 mg/kg/day on days 8–21 for total 14 days)

Given that the potent pro-apoptotic effect of midostaurin may help increase rituximab antitumor activity, we tested the efficacy of a combination treatment of rituximab with midostaurin in immunodeficient mice that were implanted with the more resistant Raji32 cells transfected with a luciferase-expressing plasmid. On day 50 after implantation, the tumor growth represented by total flux was ranked in the order of saline, rituximab, midostaurin and rituximab plus midostaurin, in which all the adjacent groups exhibited statistically significant differences in tumor mass (Fig. 6a, b). On day 70, 3 out of 7 mice died in the saline group, 1 out of 7 died in the rituximab group, and all 7 mice survived in the midostaurin and combination treatment groups (Fig. 6c). The tumor mass in the surviving mice showed consistent results (Fig. 6d). On day 90, 1 out of 7, 4 out of 7, or 6 out of 7 mice survived in the saline or rituximab groups, midostaurin group, or combination treatment group, respectively (Fig. 6e). The tumor mass in the surviving mouse with saline treatment was greater than that in the surviving mouse with rituximab treatment (Fig. 6f). In addition, the survival curve analysis demonstrated that compared to rituximab alone, midostaurin alone or in combination with rituximab significantly prolonged the survival rate (Fig. 6g), indicating that the pro-apoptotic effect induced by midostaurin may be a necessary and beneficial supplement to the therapeutic regimen, especially for the treatment of resistant BL.

## Discussion

In the present study, we revealed that PKC was highly up-regulated and activated in rituximab-resistant BL cells. The administration of the pan-PKC inhibitor midostaurin alone or in combination with rituximab potently elicited apoptosis and dramatically improved OS, especially in resistant BL cells. Although the function of PKC in cancer development remains controversial^45^, PKC inhibitors are being widely tested for the treatment of multiple types of cancer in clinical trials. In April 2017, midostaurin was approved for the treatment of AML with the FLT3 mutation and advanced SM^42,43,46^. Currently, no clinical trials for BL treatment are registered at clinicaltrials.gov, although there have been a small number of studies reporting the importance of PKC activation in BL cells^47,48^. Therefore, our findings suggest that it is imperative to perform a clinical trial of midostaurin alone or in combination with rituximab, preferably for relapsed/refractory BL patients and possibly for other relapsed/refractory NHL patients with highly activated PKC.

Rituximab has achieved great success in the treatment of a broad variety of B-cell lymphomas. Rituximab may destroy CD20-expressing lymphocytes mainly via ADCC and CDC by binding to the CD20 on the membrane^17–19^. However, resistance to rituximab remains a major challenge for relapsed/refractory patients. Approximately 50% of patients are unresponsive to rituximab treatment despite CD20 expression, and initially responsive patients eventually develop resistance to further rituximab treatment^17,18^. It may be not possible to overcome the resistance to ADCC because this resistance likely results from the intrinsic immune features of patients^20^. Therefore, the current approaches mainly focus on enhancing the efficacy of rituximab-mediated CDC, including up-regulating CD20 expression using the histone deacetylase inhibitor trichostatin A^37^ or synthetic CpG oligodeoxynucleotides^38^ and inhibiting CD59 function using a modified monoclonal antibody^39,40^ or a bacterial toxin-derived ILYd4^32^. However, these effects observed in experimental systems require further careful investigation, including an assessment of their toxicity.

Although a direct rituximab-induced cytotoxic effect on lymphocytes is somewhat controversial, numerous studies in various NHL cells have demonstrated that rituximab lacks pro-apoptotic capability. Some strategies have been implemented to sensitize rituximab therapy by combining with other agents to increase the pro-apoptotic effect. These additional agents include macromolecules such as the humanized monoclonal mapatumumab targeting TRAIL-R1^49^, the genetically engineered fusion proteins scFvRit:sFasL^50^, Apo2 ligand (Apo2L)/TRAIL (dulanermin)^51,52^ and anti-CD20-interleukin-21^53^, and small molecules, such as the selective NEDD8 activating enzyme inhibitor pevonedistat (MLN4924)^54^, the mTOR (mammalian target of rapamycin) inhibitor temsirolimus^55^, and the proteasome inhibitor bortezomib^56^. However, the efficacy of these agents requires further confirmation or has been suggested to be insufficient based on clinical trials^52^. Interestingly, obinutuzumab (GA101), a novel type II glycol-engineered humanized monoclonal anti-CD20 IgG1 antibody, has shown its superior efficacy against CLL (chronic lymphocytic leukemia) and relapsed/refractory indolent NHL to rituximab^57,58^, due to the enhanced capability of inducing direct cell death and ADCC, though less potent in mediating CDC^59^. Moreover, the similar superior efficacy of obinutuzumab to rituximab has also been observed in rituximab-sensitive/resistant BL^60,61^. Although the underlying mechanisms by which obinutuzumab is able to induce strong direct cell death remain largely obscure, several mediators have been suspected including reactive oxygen species^27^, BCR (B cell antigen receptor) or cytotoxicity pathway^61^. Therefore, whether addition of midostaurin to obinutuzumab could enhance the antitumor activity is worthy of future investigation. Herein, we reported that midostaurin strongly enhanced rituximab activity by promoting apoptosis via PKC inhibition in BL cells.

Midostaurin was modified from staurosporine to improve the selectivity toward PKC and was then further found to inhibit other kinases, such as FLT3, PDGFR, KIT and VEGFR2, together with the midostaurin metabolites^62^. Midostaurin was found to be safe and tolerable when administered as a chronic oral therapy in a phase I clinical trial^63^ and was approved by the FDA (Food and Drug Administration) based on its effectiveness^64^. Considering that PKC was highly phosphorylated in the resistant BL cells, we tested the in vitro and in vivo effects of midostaurin on potentiating rituximab antitumor activity in the original and resistant BL cells. We found that midostaurin strongly enhanced rituximab cytotoxicity, especially in the resistant BL cells, by promoting apoptosis, possibly by altering the phosphorylation of downstream signaling molecules, including Bad, Bcl-2 and NF-κB. Our findings support the further evaluation in clinical trials of midostaurin alone or in combination with rituximab and with or without short-term intensive chemotherapy in relapsed/refractory BL patients.

## Supplementary information

Table S1. the list of antibodies in this study

Supplementary Data 1

Supplementary Data 2
