# Supplementary material for: Midostaurin potentiates rituximab antitumor activity in Burkitt’s lymphoma by inducing apoptosis
Source: Cell Death Dis. 2018 Dec 18;10(1):8. doi: 10.1038/s41419-018-1259-5 (PMC6315025; doi:10.1038/s41419-018-1259-5)
Supplement: Supplementary file 1 — Table S1. the list of antibodies in this study [file 41419_2018_1259_MOESM1_ESM.docx]

Table S1. The commercial antibodies used in this study.

| **Antibodies** | **Manufacturers** | **Applications in this study** | **Catalog Number** |
| --- | --- | --- | --- |
| CD59 (H-7) | Santa Cruz Biotechnology | WB (1:500) | sc-133170 |
| CD55 (H-7) | Santa Cruz Biotechnology | WB (1:500) | sc-133220 |
| CD46 Rabbit Polyclonal antibody | Proteintech | WB (1:500) | 12494-1-AP |
| CD20 (L26) | Santa Cruz Biotechnology | WB (1:500) | sc-58985 |
| β-actin (C4) | Santa Cruz Biotechnology | WB (1:1,000) | sc-47778 |
| Phospho-Akt (Ser473) (D9E) | Cell Signaling Technology | WB (1:1,000) | 4060 |
| Akt1 (C73H10) | Cell Signaling Technology | WB (1:1,000) | 2938 |
| Phospho-NF-kB(Ser536) (93H1) Rabbit mAb | Cell Signaling  Technology | WB (1:1000) | 3033 |
| NFKB p65 (F-6) | Santa Cruz Biotechnology | WB (1:500) | sc-8008 |
| goat anti-mouse IgG-HRP | Santa Cruz Biotechnology | WB (1:10,000) | sc-2005 |
| goat anti-rabbit IgG-HRP | Santa Cruz Biotechnology | WB (1:10,000) | sc-2004 |
| PE mouse Anti-Human CD59 (H19) | BD Pharmingen | FACS (20μL/test) | 560953 |
| Hu CD55 PE (IA10) | BD Pharmingen | FACS (5μL/test) | 561901 |
| APC anti-human CD46 (TRA-2-10) | BioLegend | FACS (5μL/test) | 352405 |
| APC anti-human CD20 (2H7) | BioLegend | FACS (5μL/test) | 302310 |
| Anti-PKC alpha antibody [Y124] | abcam | WB (1:1000) | ab32376 |
| Anti-PKC beta1 antibody [EPR18512] | abcam | WB (1:1000) | ab195039 |
| Anti-PKC beta2 antibody [Y125] | abcam | WB (1:1000) | ab32026 |
| Anti-PKC gamma antibody [EPR5979(2)] | abcam | WB (1:1000) | ab131222 |
| Anti-PKC eta antibody [EPR18513] | abcam | WB (1:1000) | ab179524 |
| Anti-Bcl-2 antibody [E17] | abcam | WB (1:1000) | ab32124 |
| Phospho-Bcl-2 (Thr56)  antibody | Cell Signaling  Technology | WB (1:1000) | 2875 |
| Phospho-Bad (Ser112)  (40A9) Rabbit mAb | Cell Signaling  Technology | WB (1:1000) | 5284 |
| Bad (D24A9) Rabbit mAb | Cell Signaling  Technology | WB (1:1000) | 9239 |
